# Modernized Synthesis Technique of Pr_2_NiO_4+δ_-Based Complex Oxides Using Low-Temperature Salt Melts

**DOI:** 10.3390/ma15176148

**Published:** 2022-09-05

**Authors:** Artem P. Tarutin, Stanislav A. Baratov, Dmitry A. Medvedev

**Affiliations:** 1 Laboratory of Electrochemical Devices Based on Solid Oxide Proton Electrolytes, Institute of High, Temperature Electrochemistry, Ekaterinburg 620990, Russia; 2 Institute of Hydrogen Energy, Ural Federal University, Ekaterinburg 620002, Russia

**Keywords:** praseodymium nickelate, nitrate melt, SOFC, SOEC, electrode, layered oxides

## Abstract

Phases based on layered lanthanide nickelates are considered as promising electrode materials for various electrochemical devices, including solid oxide fuel cells and electrolysis cells. While such compounds may be prepared using either solid state or solution-assisted syntheses, each of these approaches entails certain problems. In the present work, we propose a novel approach for the simple and straightforward preparation of Pr_2_NiO_4+δ_-based materials. This approach involves co-melting of initial nitrate components, followed by high-temperature decomposition of the obtained mixture. The developed synthesis method exhibits a number of advantages over conventional techniques, enabling highly dispersed and single-phase powders to be obtained at a reduced synthesis temperature of 1050 °C. Therefore, the results of this work open new possibilities for the cost-effective preparation of Ruddlesden–Popper oxide phases.

## 1. Introduction

Lanthanide nickelate materials (Ln_2_NiO_4+δ_, Ln = La, Pr, Nd) constitute an attractive class of complex oxides for applied electrochemical purposes: as electrode materials for solid oxide fuel cells and electrolysis cells [1,2]; as components of cathode materials for lithium-ion batteries [3,4]; as oxygen permeable membranes for oxygen separation [5,6]; as efficient (photo)electrocatalysts toward oxidation [7,8] and hydrogen evolution [9,10] reactions; as components of sensors [11,12,13], etc. Such a wide range of potential applications is due to features in their layered crystal structure, which offer high ionic and electronic transport combined with relatively good chemical stability and mechanical properties, starting from standard conditions and ending with high-temperature operation at 800–1000 °C.

Ln_2_NiO_4+δ_-based materials involving various doping modifications can be synthesized via a number of synthesis routes, including the standard solid-state reaction method [14], co-precipitation [15], the sol-gel method [16], the nitrate-combustion method [17,18,19], etc. Although materials may be successfully synthesized using these techniques, each has its own inherent disadvantages. For example, in order to ensure a single-phase state of layered nickelates, the solid-state reaction method requires high temperatures and prolonged treatment times. The oxidation by nitrate groups of additional organic substances serving as a fuel for sol-gel and nitrate-combustion techniques involves the release of large quantities of gases, resulting in a highly dispersed and porous residue. The co-precipitation method requires careful control of the process conditions for the simultaneous precipitation of all required cations. In short, the multi-step or time/cost-consuming technological processes involved in the considered methods inspire a search for new convenient and technologically efficient synthesis methods.

In the present work, we present for the first time an original approach developed for the preparation of doped nickelates in single-phase forms at temperatures as low as 1050 °C. The described approach utilizes the corresponding nitrates with no additional substances required (organic, ammonia, precipitator). The further careful calcination of the homogeneous viscous solution that formed during the co-melting of nitrates resulted in highly dispersed powders forming corresponding single-phase products following twice-calcination at 1050 °C.

## 2. Materials and Methods

In the present work, two widely used synthesis routes were employed for the synthesis of Pr_2_Ni_0.9_Co_0.1_O_4+δ_ as an example: the solid-state synthesis method (SS) [20] and citrate–nitrate combustion synthesis (CN) [21]. Along with these two routes, a proposed new synthesis approach based on the decomposition of low-temperature molten salts (MS) was conducted.

In the SS method, the following compounds with a purity of 99.5% and above were used: Pr_6_O_11_, NiO and Co_3_O_4_. The required amounts of the preliminary dried powders were mixed in ethanol media for 1 h via an agate mortar and pestle. The ground powder was pressed into discs to ensure better reactivity of the components and then calcined at 1050 °C for 5 h. After grinding, the calcined samples were then again pressed for further calcination at the same temperature. These steps were repeated twice to achieve good homogeneity of the final powder.

For the CN method, Pr_6_O_11_, metallic nickel and cobalt were mixed in strictly required proportions and then dissolved in a solution of nitric acid. Citric acid was then added in a mole ratio of 1.5:1 to the total metal amount. The obtained solution was evaporated at 240 °C under constant stirring. After being brought to a higher temperature (~350 °C), the obtained substance was ignited, resulting in the formation of a residue, which was ground and calcined at 1050 °C for 5 h in a 3-step process as in the SS method.

The proposed MS method is based on mixing low-temperature nitrate melts of Pr(NO_3_)_3_·6H_2_O, Ni(NO_3_)_2_·6H_2_O and Co(NO_3_)_2_·6H_2_O, followed by their subsequent decomposition. This should result in the formation of a highly dispersed, chemically homogeneous mixture of oxides. For the formation of the target Pr_2_Ni_0.9_Co_0.1_O_4+δ_ phase, high-temperature calcination is required. Due to the low melting temperature (around 80 °C) of the used nitrates (their crystal hydrate forms), a high-quality and energy-efficient homogeneity of nitrate mixture can be achieved. The relatively simple process of obtaining a single-phase product by the calcination of this mixture additionally involves low energy consumption due to the shorter duration of high-temperature treatments due to the high degree of chemical homogeneity of the mixture. To realize the MS method, the corresponding nitrates taken in the required ratio were placed in an Al_2_O_3_ crucible and then heated at 86 °C for 10 min. After this, a homogeneous and highly viscous melt was formed. This melt, after its cooling, was calcined at 1050 °C for 5 h. As a result of the high-temperature treatment, the nitrates decomposed to form a highly dispersed ash. The calcined powder was ground and finally synthesized at 1050 °C for 5 h.

To analyze the phase quality of the obtained powders, X-ray powder diffraction (XRD) analysis was performed applying a D/MAX-2200VL/PC diffractometer (Rigaku Co., Ltd., Japan) via the Cu Kα-radiation at room temperature in ambient air in the 2θ range of 20°–80°.

The morphology and elemental composition of the powders were analyzed by scanning electron microscopy (SEM) analysis using a Phenom ProX electron microscope (Thermo Fisher Scientific, USA).

Thermogravimetric analysis (TGA) was performed using a NETZSCH STA 449 F3 Jupiter thermal analyzer in air to evaluate features of the decomposition processes for the powders prepared by all three routes. The measurements were performed from 25 to 1000 °C with a heating rate of 3 °C min^–1^ in extra pure (99.999%) argon.

## 3. Results

### 3.1. Thermal Behavior of Individual Nitrates

**Figure 1:**
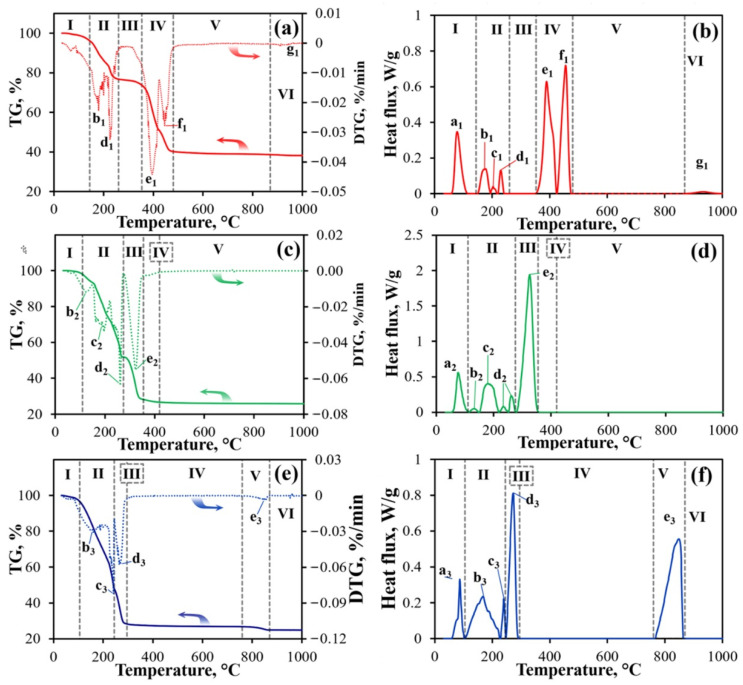
Thermal analysis of nitrate salts: TG and differential (DTG) data for Pr(NO_3_)_3_·6H_2_O (**a**), Ni(NO_3_)_2_·6H_2_O (**c**) and Co(NO_3_)_3_·6H_2_O (**e**) and corresponding DSC data (**b**), (**d**) and (**f**), respectively. The origin of the mentioned peaks can be found in Table 1.

In order to justify the MS method, it is necessary to characterize the initial nitrate salts in terms of their melting points. The experimental results and their analysis are presented in Figure 1 and Table 1. The chemical compositions of the samples and the ongoing chemical processes were evaluated by analyzing the temperature dependences of weight and heat flux.

The five sections are revealed in the TG curve of Pr(NO_3_)_3_·6H_2_O (Figure 1a). Here, Section I corresponds to the initial heating and melting of this salt [22]. Although the a_1_ peak appears at ~60 °C in the DSC curve, no weight loss is observed here. In Section II (144–260 °C), the subsequent removal of water molecules takes place (peaks b_1_, c_1_, and d_1_ [23,24]). Since Section III does not show any weight loss or thermal effects, this section can be taken to correspond to heating of the anhydrous Pr(NO_3_)_3_. With further heating, praseodymium nitrate decomposes in Section IV. According to the TG and DSC analyses, this decomposition occurs through the two stages (peaks e_1_ and f_1_ [22]). Most probably, a quasi-stable compound of PrONO_3_ could exist [23]. Then, the latter transforms to PrO_2_ [22,23]. No visible changes were found in the section V, indicating a simple heating of PrO_2_ up to 880 °C. At higher temperatures, a small weight loss can be associated with oxygen desorption from PrO_2_ coupled with the formation of Pr_2_O_3_. The modest DSC-peak associated with this process is due to the small thermal effect of this reaction.

The melting process of Ni(NO_3_)_2_·6H_2_O (peak a_2_) occurs at a temperature close to the melting point of Pr(NO_3_)_3_·6H_2_O. As evidenced by the presence of several peaks (b_2_, c_2_) on the DSC curve, the dehydration process also proceeds stepwise. Based on the analysis of the d2 peak, the dehydration process can be concluded to be accompanied by the partial decomposition of nickel nitrate to nickel nitrite. Thermal decomposition of nickel nitrite to Ni_2_O_3_ is observed at peak e2, while Ni_2_O_3_ begins to decompose to stable NiO oxide at a temperature of around 350 °C. The results obtained are consistent with those reported by other groups [25,26].

The melting point of cobalt nitrate hexahydrate, Co(NO_3_)_2_·6H_2_O (Section I), is also close to those of Ni(NO_3_)_2_·6H_2_O and Pr(NO_3_)_3_·6H_2_O (peak a_3_). In Section II (105–247 °C) of the TG curve, a rapid weight loss occurs (peak b_3_), corresponding to its full dehydration until Co(NO_3_)_2_ [27]. Section III (247–295 °C) could be related to a Co(NO_3_)_2_ → Co_3_O_4_ decomposition (peak d_3_) [26]. Apparently, oxide formation is preceded by the partial decomposition of cobalt nitrate to nitrite (peak c_3_ [27]). Since no visible transformations occurred in the temperature range of 295–760 °C (Section IV), heating of the Co_3_O_4_ oxide can proceed. With further heating (760–870 °C), the Co_3_O_4_ decomposes to CoO in section V (peak e3).

Based on the obtained results, the principal possibility of a stable melt composed of nickel, praseodymium, and cobalt nitrates exists at temperatures from 60 °C to 105 °C. For the preparation of the required melts, we selected a temperature of 85 °C as a certain compromise between melt viscosity and stability.

### 3.2. Comparison of Three Synthesis Routes

**Figure 2:**
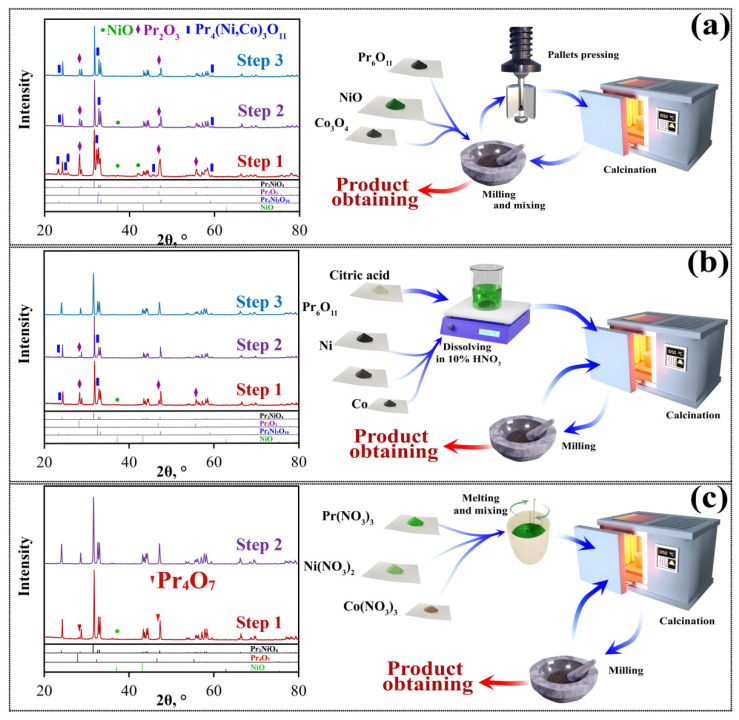
XRD results of the Pr_2_Ni_0.9_Co_0.1_O_4+δ_ powders prepared by the SS (**a**), CN (**b**) and MS (**c**) techniques followed by several calcination steps as well as the schematic representation of the used synthesis methods.

For the purposes of comparison, the same Pr_2_Ni_0.9_Co_0.1_O_4+δ_ composition obtained by the proposed MS technique was also synthesized using the SS and CN methods. Figure 2 shows the main stage for the powder preparation coupled with the XRD data. According to Figure 2a, no single-phase product is obtained in this work when utilizing the SS method. Following the first calcination, the following impurity phases were detected along with the main layered phase: Pr_6_O_11_ (~20 wt.%), Pr_4_(Ni,Co)_3_O_10_ (~22 wt.%) и NiO (~22 wt.%). The weights of these phases after the second calcination decrease down to 12, 8, and ~1 wt.%, respectively. Finally, the third calcination step resulted in the co-existence of two impurities: 8 wt.% of Pr_6_O_11_ and 6 wt.% of Pr_4_(Ni,Co)_3_O_10_. Although it is probable that the target complex oxide (Pr_2_Ni_0.9_Co_0.1_O_4+δ_) can be obtained in a single-phase form during the SS method if higher temperatures are used, elevated synthesis temperatures are undesirable due to the coarsening of thus-obtained powders.

A similar analysis was carried out for the CN-obtained powders (Figure 2b). Following the first calcination, the powder contained the target layered phase along with a number of impurities, including Pr_6_O_11_ (~21 wt.%), Pr_4_(Ni,Co)_3_O_10_ (~8 wt.%), and NiO (~10 wt.%). The second calcination resulted in a considerable decrease of impurity phase quantities to around 2.5 wt.%, 5 wt.% and 2 wt.%, respectively. Finally, the third calcination led to the formation of a pure Pr_2_Ni_0.9_Co_0.1_O_4+δ_ phase. These results confirm that the solution techniques exhibit advantages over conventional solid-state synthesis, particularly in terms of higher molecular homogenization, which requires lower temperatures to achieve single-phase products.

For the MS-prepared powder, two impurities in small amounts (~2.5 wt.% of Pr_6_O_11_ and ~0.5 wt.% of NiO) were detected following the decomposition of nitrates during the first calcination. In contrast to the two previously-described synthesis techniques, the twice-calcined powder was found to be fully single-phase (Figure 2c).

**Figure 3:**
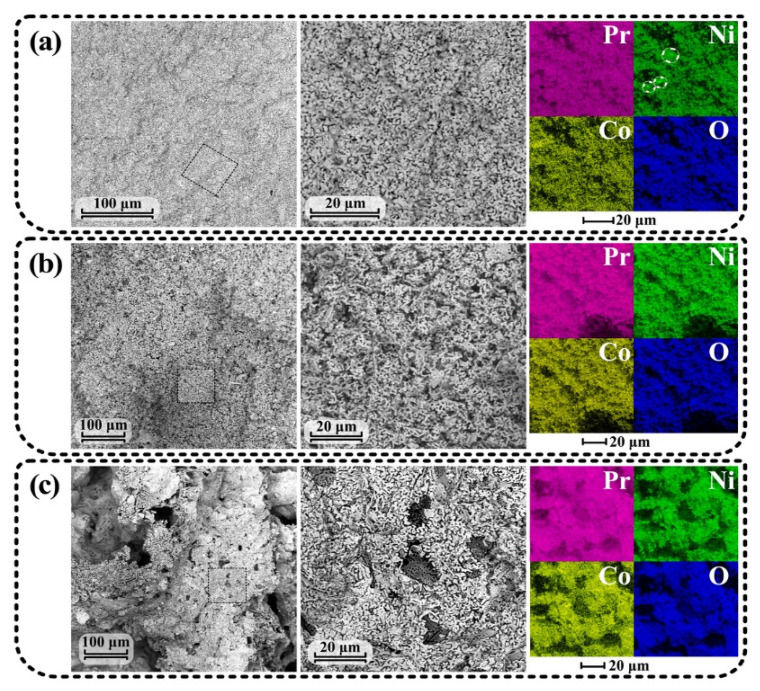
SEM and EDX analyses of the Pr_2_Ni_0.9_Co_0.1_O_4+δ_ samples prepared by the SS (**a**), CN (**b**) and MS (**c**) techniques.

The cross-section of the ceramic materials obtained by the SS, CN and MS routes after their first annealing characterized by means of SEM analysis is shown in Figure 3.

In the SEM images of the calcined sample obtained using the SS method (Figure 3a), highly porous and rather coarse particles of various oxides can be distinguished. The particle size is quite heterogeneous and varies from 0.8 to 7 µm with a median value of 1.2 µm. Element distribution maps show regions enriched with nickel (marked with circles) containing phases such as NiO and Pr_4_Ni_3_O_10_. From these facts, it can be concluded that the durations of mixing of the initial substances and isothermal exposure were insufficient.

For the sample obtained by the SS method (Figure 3b), the formation of a disordered porous structure composed of chain and flat components can be seen to have taken place. The particle size varied from 0.6 to 1.1 µm with a median value of 0.85 µm.

From the fracture of the MS-obtained sample (Figure 3c) it can be seen that the sample is highly porous. This might be explained by the release of a high quantity of gases during the decomposition of nitrates. The resulting oxide powder contained elongated grains less than 0.1 μm in thickness and from ~1 to ~4 μm in other directions. The EDX maps showed uniform element distribution, confirming the achievement of high chemical homogeneity following the first annealing step. The crystallite size calculated after the final synthesis temperature was found to be equal to ~40, 15 and 16 nm for the powders prepared by the SS, CN and MS methods, respectively. This indicates that the MS method allows the formation of highly dispersed (agglomerated) particles similarly with various solution techniques, while the SS method is characterized by more coarse particles.

Based on this analysis, it can be concluded that, despite the energy consumption of the SS synthesis method, it did not allow high chemical homogeneity of the powder to be achieved. In contrast, the SS and MS methods are free from this disadvantage. 

An important characteristic for powders used for the fabrication of SOFC electrodes is their specific surface, this being the characteristic that directly affects the resulting electrochemical activity. The specific surface area of the SS-prepared powder after first calcination was found to be equal to 4.4 m^2^ g^–1^ versus 6.2 m^2^ g^–1^ for the CN-prepared powder and 7.0 m^2^ g^–1^ for the MS-prepared powder. This allows the latter method to be considered as promising for obtaining complex oxides based on Pr_2_NiO_4+δ_.

**Figure 4:**
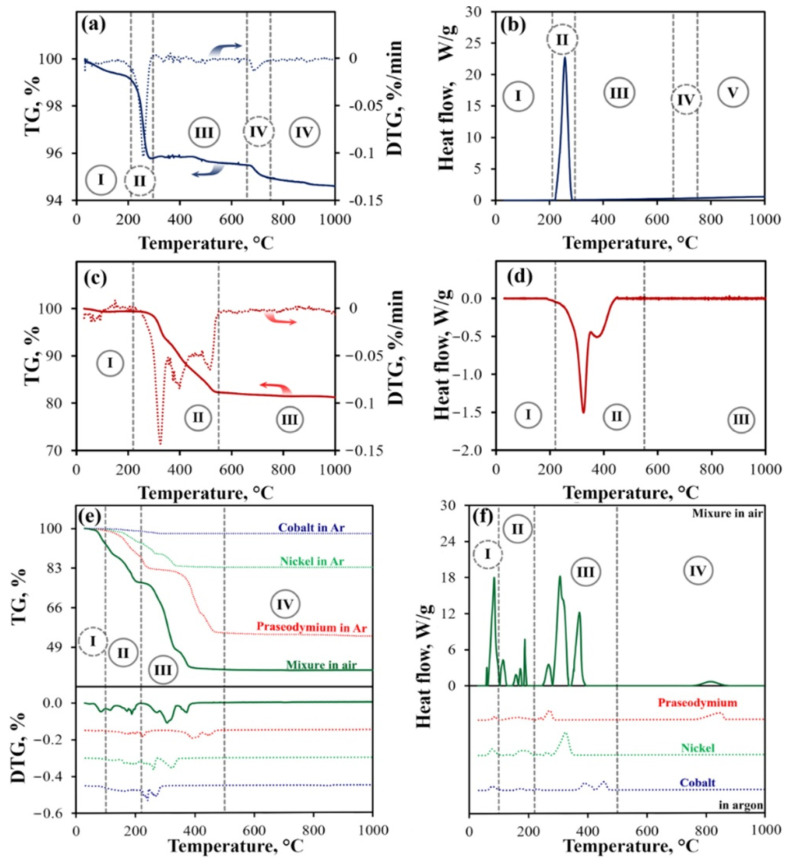
TG (**a**,**c**,**e**) and DSC (**b**,**d**,**f**) data for the samples obtained via the SS method (**a**,**b**), CN method (**c**,**d**), and MS method (**e**,**f**). The panels (**e**,**f**) provide the corresponding data for individual nitrates normalized to their weight content in the Pr_2_Ni_0.9_Co_0.1_O_4+δ_ phase.

The powders on the nominal Pr_2_Ni_0.9_Co_0.1_O_4+δ_ composition obtained after the SS, CN and MS methods were further analyzed by TG-DSC in an air-argon mixture with an oxygen partial pressure of 0.17 atm. A heating rate was equal to 1 °C min^– 1^ (Figure 4). Thus, the conditions of the first heating of these samples were partially repeated. As a result, the chemical processes occurring during high-temperature treatment were studied.

Figure 4a,b present TG, DTA, and DSC data for a mixture of oxides. There are five sections on the TG curve. Apparently, in Section I (25–210 °C), a trace of acetone added to the powder mixture during its grinding was evaporated. In Section II, the thermal decomposition of Pr_6_O_11_ to Pr_2_O_3_ evidently occurs with the release of O_2_. In Sections III–V, the formation of new phases is apparently accompanied by the removal of oxygen from the resulting structures. No significant thermal effects were found on the DSC curve.

Figure 4c,d present the corresponding results for the residue obtained following citrate–nitrate combustion. The TG and DSC curves can be separated by three sections. The weight decreases insignificantly in Section I. In all likelihood, this is associated with the fact that the residue was preliminarily treated at 350 °C (see Experimental section). A significant weight loss occurs in Section II. The DSC analysis reveals two peaks in this section in which intensities greatly differ. While the former peak can be related to the combustion of organic residues, the latter can be attributed to the decomposition of nitrates. As was established earlier, the processes of nitrate decomposition are endothermic, while the combustion of organic substances has an exothermic character. The simultaneous occurrence of two processes leads to a partial decrease in heat release in the temperature range of 330–440 °C. As can be seen from the data presented, heat release ends at the temperature of 440 °C, while mass loss is observed up to 540 °C. Evidently, this is due to a slow removal of gases from the reaction mass, which has a large number of pores.

The TG curve of the mixture of nitrates following their melting (MS method, Figure 4e,f) can be conditionally divided into 4 sections. In Section I, melting of nitrates occurs with a small weight change, but considerable heat effects. In Section II, the TG-DSC curves show complete dehydration of the mixture, which is confirmed by previous results where the reagents were studied individually (Figure 1). When the anhydrous mixture is heated above 250 °C, nitrates start to decompose. This process ends at ~400 °C (Section III), with further heating of the mixture (Section IV) resulting in no significant changes in mass or the appearance of peaks in the DSC curve. Apparently, in this temperature range, mutual diffusion of the formed oxides occurs, which is not tracked according to the TG data.

Figure 4e,f also include the data obtained for individual nitrates. It can be seen that all processes occurring for the individual nitrates are duplicated for the nitrate mixture, taking the nitrate ratio into account. A distinctive feature here is the observed decrease in temperatures for some processes associated with the dehydration of nitrates in the mixture and the decomposition of salts.

The proposed method of the joint nitrate metals’ decomposition is found to be promising for producing nickel-based layered phases. However, this method exhibits certain limitations. First, only nitrates with close melting temperatures can be used; otherwise, no homogeneous melt solution is obtained, which could prevent the subsequent formation of single-phase products. Second, nitrate decomposition is accompanied by an intense release of nitrogen oxides, NO_x_, which are acidic components having a detrimental effect on the environment. Finally, the costs of nitrates are, as a rule, higher compared to the corresponding oxides or carbonates. Nevertheless, this approach can be considered suitable for the lab-scale preparation of nano-sized powders of nickelites and other related compounds with a small quantity.

## 4. Conclusions

In the present work, a nitrate co-melting technique was successfully used for the preparation of the single-phase Pr_2_NiO_4+δ_-based materials using a relatively mild synthesis regime (twice calcination at 1050 °C for 5 h without using ball milling). This technique was shown to possess advantages over the solid-state synthesis and citrate-nitrate combustion methods, whose utilization requires higher calcination temperatures or prolonged times to obtain the desired product. Thus, the co-melting of nitrates can be considered as a promising approach for the preparation of other layered complex phases based on Ln_2_NiO_4+δ_.

## Figures and Tables

**Table 1:** The main physico-chemical steps of nitrates decomposition revealed by the TG-DSC analysis.

Processes	Peak Index	T_start_, °C	T_peak_, °C	ΔH, kJ mol^–1^
**Pr(NO_3_)_3_·6H_2_O**				
Melting of Pr(NO_3_)_3_·6H_2_O	a_1_	60	80	40.2
Pr(NO_3_)_3_·6H_2_O → Pr(NO_3_)_3_·3H_2_O+ 3H_2_O↑	b_1_	150	170	17.4
Pr(NO_3_)_3_·3H_2_O = Pr(NO_3_)_3_·2H_2_O + H_2_O↑	c_1_	195	200	2.2
Pr(NO_3_)_3_·2H_2_O = Pr(NO_3_)_3_ + 2H_2_O↑	d_1_	220	230	6.2
Pr(NO_3_)_3_ = PrONO_3_ + 2NO_2_↑ + 0.5O_2_	e_1_	350	390	66.3
PrONO_3_ = PrO_2_ + NO_2_↑	f_1_	425	455	40.3
2PrO_2_ = Pr_2_O_3_ + 0.5O_2_↑	g_1_	~880	-	-
**Ni(NO_3_)_2_·6H_2_O**				
Melting of Ni(NO_3_)_2_·6H_2_O	a_2_	60	80	40.3
Ni(NO_3_)_2_·6H_2_O = Ni(NO_3_)_2_·5H_2_O + H_2_O↑	b_2_	110	140	3.2
Ni(NO_3_)_2_·5H_2_O = Ni(NO_3_)_2_·1.5H_2_O + 3.5H_2_O↑	c_2_	150	180	51.1
Ni(NO_3_)_2_·1.5H_2_O = Ni(NO_2_)_2_ + O_2_↑ + 1.5H_2_O↑	d_2_	220	230	9.2
2Ni(NO_2_)_2_ = Ni_2_O_3_ + 4NO + 0.5O_2_	e_2_	280	330	85.3
Ni_2_O_3_ = 2NiO + 0.5O_2_		350	-	-
**Co(NO_3_)_2_·6H_2_O**				
Melting of Co(NO_3_)_2_·6H_2_O	a_3_	60	90	5.6
Co(NO_3_)_2_·6H_2_O = Co(NO_3_)_2_ + 6H_2_O↑	b_3_	100	170	14.8
Co(NO_3_)_2_ = Co(NO_2_)_2_ + O_2_↑	c_3_	230	240	1.4
3Co(NO_2_)_2_ = Co_3_O_4_ + 2NO_2_↑ + 4NO↑	d_3_	250	270	7.8
Co_3_O_4_ = 3CoO + 0.5O_2_	e_3_	765	850	9.8

## Data Availability

Not applicable.

## References

[1] Tarutin A.P., Lyagaeva J.G., Medvedev D.A., Bi L., Yaremchenko A.A. (2021). Recent advances in layered Ln_2_NiO_4+δ_ nickelates: Fundamentals and prospects of their applications in protonic ceramic fuel and electrolysis cells. J. Mater. Chem. A.

[2] Ishihara T. (2014). Oxide ion conductivity in defect perovskite, Pr_2_NiO_4_ and its application for solid oxide fuel cells. J. Ceram. Soc. Jpn..

[3] Ge H.-L., Wang Z.-Y., Li G.-R., Liu S., Gao X.-P. (2022). La_2_NiO_4_ nanoparticles as a core host of sulfur to enhance cathode volumetric capacity for lithium–sulfur battery. Electrochim. Acta.

[4] Nowroozi M.A., Wissel K., Donzelli M., Hosseinpourkahvaz N., Plana-Ruiz S., Kolb U., Schoch R., Bauer M., Malik A.M., Rohrer J. (2020). High cycle life all-solid-state fluoride ion battery with La_2_NiO_4+δ_ high voltage cathode. Commun. Mater..

[5] Chen T., Xu Y., Zhang Y., Gong Y., Zhang Y., Lin J.Y.S. (2022). Double-layer ceramic-carbonate hollow fiber membrane with superior mechanical strength for CO2 separation. J. Membr. Sci..

[6] Han N., Guo X., Cheng J., Liu P., Zhang S., Huang S., Rowles M.R., Fransaer J., Liu S. (2021). Inhibiting in situ phase transition in Ruddlesden-Popper perovskite via tailoring bond hybridization and its application in oxygen permeation. Matter.

[7] Tao Y., Chen L., Ma Z., Zhang C., Zhang Y., Zhang D., Pan D., Wu J., Li G. (2022). Near-infrared-driven photoelectrocatalytic oxidation of urea on La-Ni-based perovskites. Chem. Eng. J..

[8] Amira S., Ferkhi M., Khaled A., Pireaux J.-J. (2022). Electrochemical properties of La_2_BO_4+δ_/C electrocatalysts and study of the mechanism of the oxygen reduction reaction in alkaline medium. J. Iran. Chem. Soc..

[9] Ma X., Gao Y., Yang B., Lou X., Huang J., Ma L., Jing D. (2022). Enhanced charge separation in La_2_NiO_4_ nanoplates by coupled piezocatalysis and photocatalysis for efficient H2 evolution. Nanoscale.

[10] Ma X., Luo B., Zeng Z., Hu S., Jing D. (2021). Significantly Enhanced Photocatalytic Hydrogen Generation over a 2D/2D Z-Scheme La_2_NiO_4_/g-C_3_N_4_ Hybrid Free of Noble Metal Cocatalyst. ACS Appl. Energy Mater..

[11] Zine A., Ferkhi M., Khaled A., Savan E.K. (2022). A_2_BO_4±δ_ as New Materials for Electrocatalytic Detection of Paracetamol and Diclofenac Drugs. Electrocatalysis.

[12] Vinothkumar V., Koventhan C., Chen S.-M., Huang Y.-F. (2022). A facile development of rare earth neodymium nickelate nanoparticles for selective electrochemical determination of antipsychotic drug prochlorperazine. J. Ind. Eng. Chem..

[13] Li Y., Li S., Meng W., Dai L., Wang L. (2021). Layered perovskite oxides La_n+1_Ni_n_O_3n+1_ (n = 1, 2, and 3) for detecting ammonia under high temperature. Sens. Actuators B Chem..

[14] Garali M., Kahlaoui M., Mohammed B., Mater A., Ben Azouz C., Chefi C. (2019). Synthesis, characterization and electrochemical properties of La_2–x_Eu_x_NiO_4+δ_ Ruddlesden-Popper-type layered nickelates as cathode materials for SOFC applications. Int. J. Hydrog. Energy.

[15] Haddadnezhad M., Babaei A., Molaei M.J., Ataie A. (2020). Synthesis and characterization of lanthanum nickelate nanoparticles with Rudllesden-Popper crystal structure for cathode materials of solid oxide fuel cells. J. Ultrafine Grained Nanostruct. Mater..

[16] Boumaza S., Brahimi R., Boudjellal L., Belhadi A., Trari M. (2020). Photoelectrochemical study of La_2_NiO_4_ synthesized using citrate sol gel method—application for hydrogen photo-production. J. Solid State Electrochem..

[17] Pikalova E., Kolchugin A., Zakharchuk K., Boiba D., Tsvinkinberg V., Filonova E., Khrustov A., Yaremchenko A. (2021). Mixed ionic-electronic conductivity, phase stability and electrochemical activity of Gd-substituted La_2_NiO_4_+δ as oxygen electrode material for solid oxide fuel/electrolysis cells. Int. J. Hydrogen Energy.

[18] Pikalova E., Sadykov V., Sadovskaya E., Yeremeev N., Kolchugin A., Shmakov A., Vinokurov Z., Mishchenko D., Filonova E., Belyaev V. (2021). Correlation between Structural and Transport Properties of Ca-Doped La Nickelates and Their Electrochemical Performance. Crystals.

[19] Tsvinkinberg V.A., Tolkacheva A.S., Filonova E.A., Gyrdasova O.I., Pikalov S.M., Vorotnikov V.A., Vylkov A.I., Moskalenko N.I., Pikalova E.Y. (2021). Structure, thermal expansion and electrical conductivity of La_2__–x_Gd_x_NiO_4+__δ_ (0.0 ≤x≤ 0.6) cathode materials for SOFC applications. J. Alloys Compd..

[20] Hyodo J., Tominaga K., Ju Y.-W., Ida S., Ishihara T. (2014). Electrical conductivity and oxygen diffusivity in Cu- and Ga-doped Pr_2_NiO_4_. Solid State Ion..

[21] Ogier T., Prestipino C., Figueroa S., Mauvy F., Mougin J., Grenier J.C., Demourgues A., Bassat J.M. (2019). In-situ study of cationic oxidation states in Pr_2_NiO_4+δ_ using X-ray absorption near-edge spectroscopy. Chem. Phys. Lett..

[22] Melnikov P., Arkhangelsky I.V., Nascimento V.A., Oliveira L.C.S., Guimaraes W.R., Zanoni L.Z. (2018). Thermal decomposition of praseodymium nitrate hexahydrate Pr(NO_3_)_3_·6H_2_O. J. Therm. Anal. Calorim..

[23] Hussein G.A.M., Balboul B.A.A., A-Warith M.A., Othman A.G.M. (2001). Thermal genesis course and characterization of praseodymium oxide from praseodymium nitrate hydrate. Thermochim. Acta.

[24] Strydom C.A., Van Vuuren C.P.J. (1988). The thermal decomposition of lanthanum(III), praseodymium(III) and europium(III) nitrates. Thermochim. Acta.

[25] Mikuli E., Migdal-Mikuli A., Chyzy R., Grad B., Dziembaj R. (2001). Melting and thermal decomposition of [Ni(H_2_O)_6_](NO_3_)_2_. Thermochim. Acta.

[26] Paulik F., Paulik J., Arnold M. (1987). Investigation of the phase diagram for the system Ni(NO_3_)_2_-H_2_O and examination of the decomposition of Ni(NO_3_)_2_·6H_2_O. Thermochim. Acta.

[27] Malecki A., Malecka A., Gajerski R., Prochowska-Klisch B., Podgorecka A. (1988). The mechanism of thermal decomposition of Co(NO_3_)_2_·2H_2_O. J. Therm. Anal..

